# The Mouse Primary Visual Cortex Is a Site of Production and Sensitivity to Estrogens

**DOI:** 10.1371/journal.pone.0020400

**Published:** 2011-05-24

**Authors:** Jin-Kwon Jeong, Liisa A. Tremere, Kaiping Burrows, Ania K. Majewska, Raphael Pinaud

**Affiliations:** 1 Department of Physiology, University of Oklahoma Health Sciences Center, Oklahoma City, Oklahoma, United States of America; 2 Department of Geriatric Medicine, University of Oklahoma Health Sciences Center, Oklahoma City, Oklahoma, United States of America; 3 Reynolds Oklahoma Center on Aging, University of Oklahoma Health Sciences Center, Oklahoma City, Oklahoma, United States of America; 4 Department of Neurobiology and Anatomy, University of Rochester, Rochester, New York, United States of America; 5 Center for Visual Science, University of Rochester, Rochester, New York, United States of America; New Mexico State University, United States of America

## Abstract

The classic female estrogen, 17β-estradiol (E2), has been repeatedly shown to affect the perceptual processing of visual cues. Although gonadal E2 has often been thought to influence these processes, the possibility that central visual processing may be modulated by brain-generated hormone has not been explored. Here we show that estrogen-associated circuits are highly prevalent in the mouse primary visual cortex (V1). Specifically, we cloned aromatase, a marker for estrogen-producing neurons, and the classic estrogen receptors (ERs) ERα and ERβ, as markers for estrogen-responsive neurons, and conducted a detailed expression analysis via in-situ hybridization. We found that both monocular and binocular V1 are highly enriched in aromatase- and ER-positive neurons, indicating that V1 is a site of production and sensitivity to estrogens. Using double-fluorescence in-situ hybridization, we reveal the neurochemical identity of estrogen-producing and -sensitive cells in V1, and demonstrate that they constitute a heterogeneous neuronal population. We further show that visual experience engages a large population of aromatase-positive neurons and, to a lesser extent, ER-expressing neurons, suggesting that E2 levels may be locally regulated by visual input in V1. Interestingly, acute episodes of visual experience do not affect the density or distribution of estrogen-associated circuits. Finally, we show that adult mice dark-reared from birth also exhibit normal distribution of aromatase and ERs throughout V1, suggesting that the implementation and maintenance of estrogen-associated circuits is independent of visual experience. Our findings demonstrate that the adult V1 is a site of production and sensitivity to estrogens, and suggest that locally-produced E2 may shape visual cortical processing.

## Introduction

The classic female hormone 17β-estradiol (E2), has traditionally been thought of as a steroid hormone secreted by the gonads to implement reproduction-associated behaviors through the binding to intracellular estrogen receptors that, when activated, act as transcriptional regulators and, consequently, modulate gene expression. It has been clear for decades now, however, that estrogen signaling is significantly more ubiquitous and far reaching. E2 can be produced by an array of tissues, especially the brain, and can exert local and rapid signaling that impacts many different systems through both genomic and non-genomic mechanisms [1], [2], [3], [4], [5]. For example, E2 affects brain processes that support pain sensitivity and regulates cognitive processes including learning and memory formation [1], [6], [7], [8].

Several lines of evidence also suggest that estrogenic signaling can affect visual processing. For instance, the perceptual processing of visual cues, including faces, and performance in visual memory tasks, positively and strongly correlate with E2 levels through the menstrual cycle in women [9], [10], [11], [12]. In addition, women with Turner syndrome, who are deficient in E2, exhibit profound deficits in visual function, including abnormal spatiotemporal processing and deficits in object perception tasks [13], [14]. Finally, post-menopausal women subjected to estrogen replacement therapy perform better on visual memory tasks than untreated women, suggesting that E2 levels may directly affect visual function [15].

These findings suggest that the visual system, in particular the visual cortex, may be a site that is influenced by E2. It is unclear, however, if estrogen-sensitive neurons are present within visual cortical circuitry. It is also unknown if hormone derived from the gonads is the only potential source of E2 to visual neurons, or whether brain-generated (neuro)hormone could potentially influence visual cortical cells. The possibility that locally-generated E2 may affect visual cortical processing is compatible with recent findings indicating that E2 is rapidly produced by central auditory neurons in an activity-dependent fashion [16] and regulates multiple aspects of sensory coding in real-time, through non-genomic mechanisms that impinge upon fast neurotransmission [17], [18].

Here we set out to directly determine if estrogen-associated circuits are present in visual cortical circuitry and, consequently, whether the occurrence of estrogenic networks is a common feature of sensory cortical areas in the vertebrate brain. Remarkably, we show that neurons in the rodent primary visual cortex (V1) express the machinery necessary for local estrogen production and sensitivity. Specifically, a vast population of V1 neurons expresses the estrogen-synthetic enzyme aromatase, and each of the classic estrogen receptors (ERα and ERβ). We also determined the neurochemical identity of estrogen-producing and estrogen-sensitive cells in V1 and discovered that these neuronal populations are heterogenous. Lastly, we determined that estrogen-associated circuits in V1 are highly stable in response to either acute or chronic manipulations of visual experience. Our results provide the first demonstration of the robust presence of estrogen-associated networks in the visual cortex. Furthermore, our findings suggest that visual processing is likely sensitive to local estrogen signaling in a manner highly analogous to the auditory system. Finally, our results suggest that local estrogen production and sensitivity may be a general mechanism for modulating cortical processing of sensory information in the vertebrate brain.

## Materials and Methods

### Animals

A total of 32 C57BL/6 mice were used in our studies. All animals were bred and housed in our vivariums at the University of Oklahoma Health Sciences Center or at the University of Rochester. The University of Oklahoma Institutional Animal Care and Use Committee (protocol # 10-093), and the University of Rochester Committee on Animal Resources (protocol # 2008-049 and # 2008-111), approved all animal use protocols. These protocols were also in full agreement with animal experimentation standards set forth by the NIH. We did not detect sex, inter-hemispheric or monocular versus binocular differences in any of the parameters evaluated in our studies. Data was, therefore, combined across sexes, hemispheres and V1 subdomains.

### Acute Visual Stimulation Studies

Before segregation into experimental groups, all mice were maintained on a 12 h light∶12 h dark cycle. The day before the experiment, animals were individually housed in standard laboratory cages and kept in a dark room overnight. The following day, mice were either killed in the dark (unstimulated controls; n = 6), or were stimulated with ambient light (luminance: 5.7 fL) for 30 min (n = 6), 1 h (n = 6) or 2 h (n = 6). We and others have repeatedly shown that this stimulation protocol drives robust expression of the activity-dependent immediate early gene early growth response-1 (*egr-1*; a.k.a., *zif268*, *NGFI-A*, *zenk* and *krox-24*) in the V1 of a variety of vertebrate species, including rodents [19], [20], [21], [22], [23].

### Chronic Visual Deprivation Studies

For chronic visual deprivation experiments, we placed four pregnant females in the dark room approximately 48 hours prior to parturition. A total of 8 animals from these four independent litters (n = 4 males and n = 4 females) were used in this study. Animals were kept in the dark-room, with their mothers, until they reached adulthood (19–20 weeks old). In short, these animals were never exposed to light. Upon reaching the appropriate age, animals were killed and brains processed, as detailed below.

### Tissue Preparation

After reaching group criteria, animals subjected to either acute stimulation or chronic deprivation studies were decapitated and brains were extracted, included in embedding medium (Tissue-Tek; Sakura Finetek, Torrance, CA), rapidly frozen in a dry-ice/ethanol bath and transferred to a −80°C freezer. For unbiased stereological analysis of both single- and double-labeled neurons, we conducted systematic-random samples of brain sections collected throughout V1. Brains, therefore, were cut at the coronal plane on a cryostat, at 40 µm thickness, were thaw-mounted on Superfrost Plus slides (Fisher Scientific, Pittsburgh, PA) and kept at −80°C until processed. Sections that were adjacent to those directed for single- or double-labeling approaches were processed for Cresyl-violet histochemistry, for definition of cortical layer boundaries based on standard cytoarchitectonic criteria [20].

### Fluorescence In-Situ Hybridization (FISH)

For our studies, we intentionally avoided the use of antibodies directed against ARO or each of the ERs. Our decision was based on the known difficulties associated with obtaining specific signals with anti-estrogen receptor (ER) antibodies. For instance, an array of antibodies directed against ERs may yield single-bands in western-blot analyses, giving the impression of specificity, but these antisera detect the same bands in tissue obtained from animals that are deficient in ERs (ER knock-outs). Additionally, several brain cell groups recognized by ER antisera are also detected in the brains of ER knock out mice, further highlighting difficulties with these antibodies. In fact, a recent study has conducted systematic comparisons of several ER antisera and revealed pitfalls and specificity limitations in using this approach [24]. In order to avoid these specificity issues, we recently cloned the mouse ARO, ERα and ERβ genes from a cDNA library, via PCR, and utilized these cDNAs to produce antisense riboprobes for in-situ hybridization to specifically and selectively detect estrogen-producing and estrogen sensitive neurons in V1. In-situ hybridization is a notoriously selective and sensitive approach to map the functional and neurochemical organization of brain circuits. Moreover, our group has recently developed, and successfully used, protocols for fluorescence in-situ hybridization that yield reliable mRNA detection, with single cell resolution in fresh-frozen brain sections [17], [25], [26]. Finally, riboprobes generated with our cloned ARO, ERα and ERβ clones selectively and specifically recognize the mRNAs encoded by each of these genes, as assessed by stringent northern-blot analyses (data not shown).

#### Riboprobe synthesis via in-vitro transcription

We used the QIAprep Spin miniprep kit (QIAGEN Inc., Valencia, CA) to purify plasmids containing: 1) ARO, the estrogen-synthetic enzyme and a marker for estrogen-producing cells; 2) ERα and 3) ERβ, the classic estrogen receptors and markers for estrogen-responsive cells; 4) *egr-1*, an activity-dependent early gene, and a reliable marker for visually-driven neurons; 5) *vGlut2*, the vesicular glutamate transporter 2, and a classic marker for excitatory neurons; 6) GAD65, one of the synthetic enzymes for GABA, and a standard marker for inhibitory neurons. Plasmids were linearized or excised by incubation with adequate restriction enzymes and inserts were purified with QIAquick PCR Purification Kit (QIAGEN Inc., Valencia, CA). Inserts were used as templates for the generation of sense and antisense riboprobes for each of the genes above, through in-vitro transcription, using protocols developed and described in detail by our group previously [17], [25], [26]. Briefly, we synthesized riboprobes using a nucleotide labeling mix containing digoxigenin (DIG)-labeled uridine triphosphate (UTP; Roche Diagnostics Corp.). Sense and antisense riboprobes were purified in Sephadex G-50 columns and 1 ng/µl of purified probe was added to 16 µl of hybridization buffer (50% formamide, 2×SSPE, 1 µg/µl BSA, 1 µg/µl poly A, 2 µg/µl tRNA in DEPC-treated water) and used for each brain section.

#### Hybridization Protocol

We have described our FISH protocols in extensive detail previously [17], [25], [26]. Briefly, sections were fixed for 5 min in a 3% paraformaldehyde solution in 0.1 M PBS, washed (3×10 min in 0.1 M PBS) and dehydrated in a standard alcohol series. Sections were incubated in acetylation solution, which consisted of 1.35% triethanolamine and 0.25% acetic anhydride in DEPC-treated water for 10 min. Subsequently tissue was rinsed in 2×SSPE, dehydrated once again, allowed to air-dry and was incubated in hybridization solution containing our riboprobe(s) of interest, as detailed above. Sections were then coversliped, sealed in a mineral oil bath and incubated at 65°C overnight. The next day, mineral oil was removed by rinsing slides in chloroform and slides were decoversliped in 2×SSPE. Tissue was then sequentially washed for 1 h in 2×SSPE (in room temperature [RT]), 1.5 h in 2×SSPE+50% formamide and 30 min in 0.1×SSPE at 65°C. Sections were then incubated in 0.3% hydrogen peroxide in TNT buffer, which consisted of 0.1 M Tris-HCl, pH = 7.4, 0.05% Triton-X 100 and 5 M NaCl in DEPC-treated water (3×10 min). Next, sections were blocked in TNB buffer, which consisted of TNT buffer+2 mg/ml BSA (30 min) and in an HRP-coupled anti-DIG antibody solution (1∶200 in TNB, Roche Dianostics Corp., Indianapolis, IN, USA; 2 h at RT). Sections were then washed in TNB (3×10 min) and incubated in tyramide-coupled Alexa 488 for 30 min (1∶200 in amplification buffer provided by manufacturer; Invitrogen, Carlsbad, CA, USA). Next, tissue was exposed to the nuclear marker Hoechst (1∶1000 in TNT buffer), washed in TNT buffer (3×10 min at RT) and coversliped with Aquamount (Lerner Labs, Pittsburgh, PA, USA). For single FISH experiments, we ran controls that included hybridization of the sense strand and omission of the anti-DIG antibody. No signal was detectable with either approach, for any of the mRNAs studied in the present work.

### Double FISH (dFISH)

We previously developed, used and detailed a dFISH protocol that allows for the simultaneous detection of two mRNAs, at single cell resolution, in frozen brain sections [25], [26]. Briefly, antisense riboprobes of interest were co-hybridized in each brain section (e.g., *egr-1* and ERα, or *vGlut2* and ARO). For each double-labeling combination, one riboprobe was labeled with DIG, as above for single FISH, and the other riboprobe was labeled with biotin, which was assembled via in-vitro transcription using biotin-tagged UTP (Roche Diagnostics Corp.). Tissue was hybridized and washed, as above, and then incubated in a solution containing HRP-conjugated anti-biotin antibody (Vector Labs, Burlingame, CA, USA; 1∶300 in TNT buffer for 2 hr at RT). Subsequently, sections were incubated in a solution containing tyramide-coupled with Alexa 594 (1∶500 in TNT buffer for 45 min at RT). We inactivated the peroxidase activity associated with the biotin-labeled riboprobe by incubating sections in 0.3% hydrogen peroxide (20 min at RT) and detected the second riboprobe by sequentially incubating sections in: 1) an HRP-conjugated anti-DIG antibody solution (1∶100 in TNB for 2 h at RT); 2) tyramide coupled to Alexa 488 (1∶200 in amplification buffer provided by the manufacturer, for 45 min at RT); 3) TNB (3×5 min at RT), 4) Hoechst (1∶1000 in TNT for 5 min at RT) and 5) TNT (3×10 min). Finally, brain sections were coversliped with Aquamount (Lerner Labs, Pittsburgh, PA, USA). Importantly, we carried out an array of controls for our dFISH studies, which have been carefully detailed previously [25], [26]. These include the use of reverse combination of tyramide reagents, which did not yield qualitative or quantitative differences in our results. We also controlled the effectiveness of the peroxidase inactivation between riboprobe detections by carrying out additional dFISH reactions where we omitted the anti-DIG antibody but incubated sections with the second Alexa substrate. These incubations just revealed signal in the adequate filter. Finally, we omitted the anti-biotin antibody in control sections to verify the specificity of the biotin labeling, as detailed previously [25], [26].

### Unbiased Stereological Quantification and Statistical Analysis

We used the optical fractionator method in a single reference space (V1) to estimate the number of cells that were positive for each riboprobe of interest. Cells were counted on an Olympus AX-70 microscope that was fitted with adequate filters, Neurolucida software (MicroBrightField) and a motorized stage. We outlined the boundaries of V1 for each section using a 4× objective and counted cells that were positive for each riboprobe with a 63× objective. A guard volume of 2 µm was used to avoid artifacts on the sliced surface of the brain sections. We used the following sampling fractions to quantify the total number of labeled cells per unit area: thickness sampling fraction (height of dissector divided by thickness of the section), section sampling fraction (number of sections sampled divided by total number of sections) and area sampling fraction (area of sampling frame divided by area of the x-y sampling step). To meet counting criteria, cells had to exhibit at least two-thirds of a cytoplasmic continuum clearly labeled around the nucleus, and unlabeled cell nucleus (i.e., “doughnut”-shaped cells). Furthermore cells were only counted if they displayed clearly defined nucleolus, as visualized with Hoechst counterstaining.

We used Hoechst staining to calculate the percentages of cells labeled for each riboprobe relative to the total neuronal density per unit area. When visualized under the adequate filter, Hoechst-labeled neurons display lightly, heterogeneously stained nuclei and prominent nucleoli. In contrast, glial cells typically exhibit strong, homogeneously stained nuclei. Neurons can be readily identified with this counterstaining even within cell clusters. Importantly, neuronal numerical densities obtained in our preparations were not significantly different from those obtained with Nissl-stained sections. We obtained group means by averaging the values obtained for each animal, and compared them using a standard analysis of variance (ANOVA) and Tukey-Kramer post-hoc tests. Significance criterion was set at p<0.05.

### Imaging and Photomicrography

Low power photomicrographs were obtained with a Nikon TE2000-E or an Olympus AX-70 epifluorescence microscope coupled to a Nikon Photometrics Cool Snap ES digital camera. High power images were obtained with a Leica SP2 MP confocal microscope. Adobe Photoshop software was used for the assembly of figure plates.

## Results

We recently demonstrated that brain-generated E2 controls the gain of central auditory neurons, in real-time, by directly regulating fast neurotransmission [17]. To determine whether estrogen-associated circuits may influence visual processing, we first assessed whether the cellular components required for estrogen production and sensitivity are available in the visual cortex, more specifically in the mouse V1. To this end, we first cloned the genes encoding aromatase (ARO; a.k.a., estrogen-synthase), and each of the classic estrogen receptors (ERα and ERβ), from a mouse cDNA library via PCR. We then used these cDNAs to generate antisense riboprobes and carried out a detailed analysis by fluorescence in-situ hybridization to determine whether or not estrogen-associated circuits are found in V1. In addition, given that this method enabled us to specifically identify estrogen-producing and estrogen-sensitive cells at single cell resolution, it was possible to quantitatively study these neuronal populations with stringent, unbiased stereological methods.

### V1 is Highly Enriched with Estrogen-Producing and Estrogen-Sensitive Neurons

**Figure 1:**
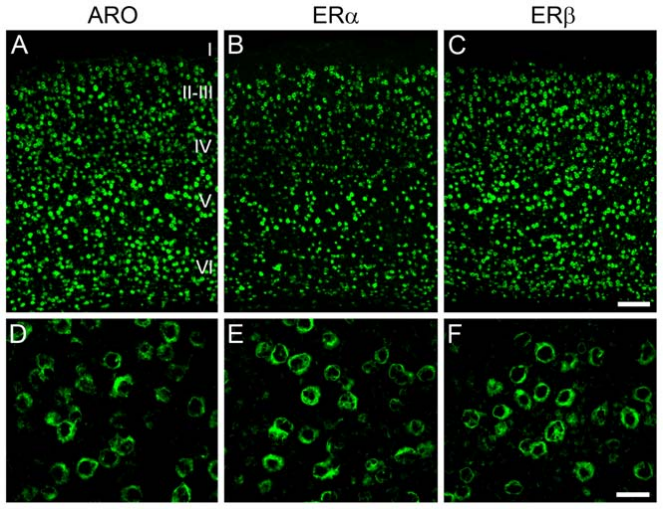
The primary visual cortex (V1) contains a large population of estrogen-producing and estrogen-producing neurons. A–C) Low-power photomicrographs depicting fluorescence in-situ hybridization directed against the mRNAs encoded by ARO (A), ERα (D) and ERβ (C) in V1. Each of these mRNAs is expressed at relatively high levels in all cortical laminae, except for layer I. D–F) Representative high power photomicrographs illustrating the labeling pattern for ARO (D), ERα (E) and ERβ (F) in the supragranular layers (II/III) of V1. The labeling pattern in the granular and infragranular layers was identical to that detected in the supragranular layers and, consequently, is not shown here. In-situ hybridization conducted with sense strand riboprobes did not reveal labeling for any of our genes of interest (data not shown). Photomicrographs were obtained with epifluorescence (A–C) and confocal (D–F) microscopy. Scale bars: A–C = 100 µm; D–F = 25 µm.

**Figure 2:**
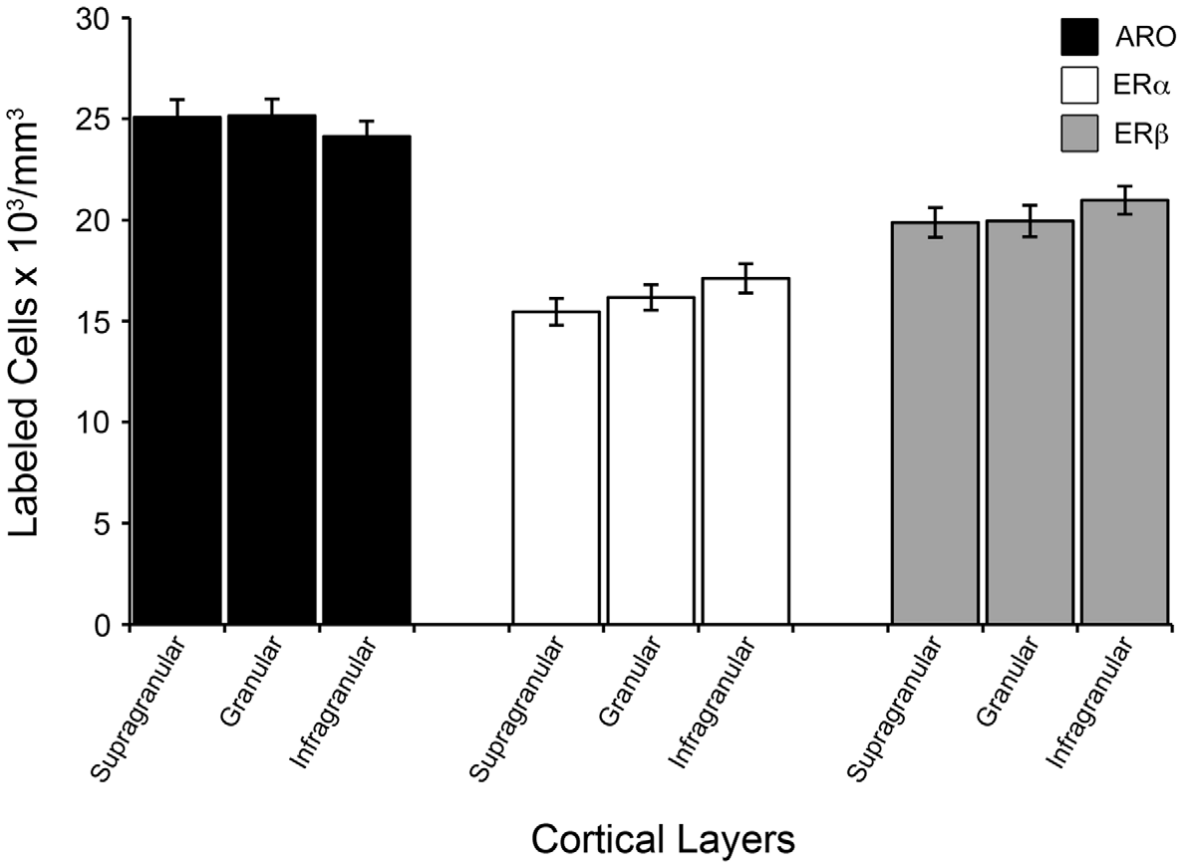
Large populations of neurons in the adult V1 putatively produce and are sensitive to estrogens. Shown are mean (± S.E.) numerical densities obtained for supragranular, granular and infragranular layers, of V1 neurons that are positive for ARO-, ERα and ERβ. Stringent unbiased stereological methods were used to obtain these values (see Methods). No statistically significant effects were detected across layers for each of the mRNAs studied.

Remarkably, we found that V1 is highly enriched in estrogen-producing (ARO-positive) and estrogen-responsive neurons (ER-positive) (Fig. 1). More specifically, we found that ARO-positive neurons are expressed at high levels in cortical layers II to VI (Fig. 1A, D). Within and across cortical layers, the distribution of cells positive for ARO mRNA was largely homogenous. Stereological quantification revealed that the supragranular (II/III), granular (IV) and infragranular (V/VI) layers of V1 contained 25.1±0.9 (mean ± S.E.), 25.2±0.8 and 24.1±0.7×10^3^ neurons/mm^3^ that were positive for ARO, respectively (Fig. 2).

V1 neurons also express both estrogen receptors abundantly. We found that ERα and ERβ are expressed in all cortical layers, with the exception of layer I (Fig. 1B–C, E–F). Although ERα is expressed at significant levels, expression of ERβ is more robust, as revealed by unbiased quantification. In particular, we determined that 15.4±0.6, 16.2±0.6 and 17.1±0.7×10^3^ neurons/mm^3^ were positive for ERα mRNA in the supragranular, granular and infragranular layers of V1, respectively (Fig. 2). Quantification of the population of ERβ-positive neurons revealed 19.8±0.7, 19.9±0.8 and 20.9±0.7×10^3^ neurons/mm^3^ in layers II/III, IV and V/VI, respectively (Fig. 2). When considering all cortical layers combined, our results showed that 63.0%±0.4, 41.3%±0.5 and 51.5%±0.8 of the overall neuronal population in V1 expresses ARO, ERα and ERβ, respectively, indicating that V1 is a major site associated with estrogenic circuits.

### Estrogen-Associated Networks in V1 Are Activated by Visual Experience

**Figure 3:**
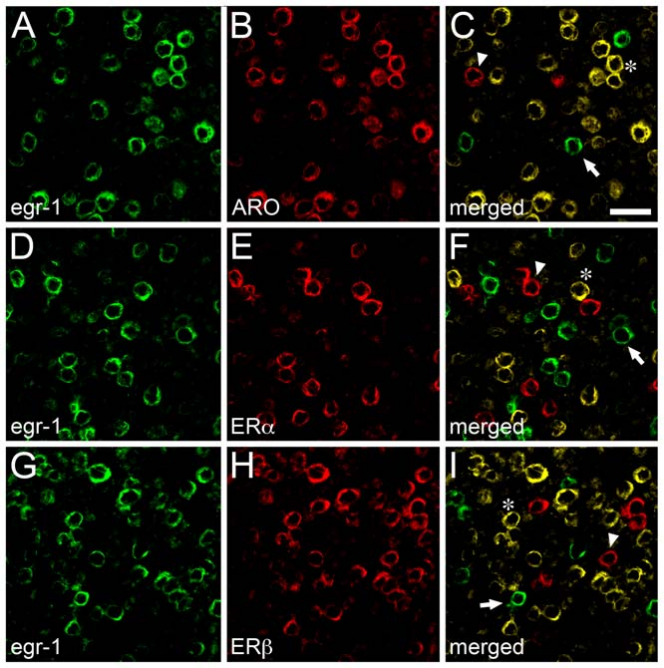
Visual experience activates estrogen-associated networks in V1. Photomicrographs illustrating the pattern of double-fluorescence in-situ hybridization (dFISH) signal in the V1 of mice stimulated for 30 min with ambient light, following overnight dark-adaptation (see Methods). Shown are representative fields depicting neurons that co-express the activity-dependent immediate early gene *egr-1* (A, D and G) and ARO (B), ERα (E) or ERβ (H) mRNAs. Merged images are shown in the right-most panels (C, F and I). Representative neurons that are positive only for *egr-1* (arrows), or exclusively for ARO, ERα or ERβ (arrowheads) can be readily identified in the images, along with examples of double-labeled neurons (asterisks). All images were obtained within a single optical slice using confocal microscopy. Scale bar = 25 µm.

We next tested if estrogen-associated circuits in V1 are engaged by visual stimulation. To this end, we housed animals overnight in a dark-room and subsequently stimulated mice with ambient light for 30 min. The V1 was then processed for a stringent double-fluorescence in-situ hybridization method that we developed and described in detail previously, where it is possible to identify two mRNAs in the same brain sections, at single-cell resolution [25], [26]. We used the expression of the activity-dependent transcription factor *egr-1* to identify visually-driven neurons in V1, and riboprobes directed against ARO or each of the ERs, to identify estrogen-producing and -responsive neurons (Fig. 3). The expression of *egr-1* has been used by a large contingent of research groups, including our own, to reliably identify visually-driven neurons, and the 30 min time-point was chosen as it corresponds to peak *egr-1* mRNA accumulation following stimulus onset (for reviews, see [19], [21], [22], [23]).

Quantitative analyses revealed that 21.8±0.8, 20.5±0.7 and 22.4±0.9×10^3^ neurons/mm^3^ in V1 co-localize *egr-1* and ARO mRNAs in supragranular, granular and infragranular layers, respectively. When considering all layers together, these results indicate that 87.3%±3.4 of visually-driven neurons are estrogen-producing cells. In contrast, we found that 4.7±0.7, 5.1±0.6 and 4.4±0.8×10^3^ neurons/mm^3^ co-localize *egr-1* and ERα mRNAs, and 12.1±0.8, 11.7±0.8 and 10.5±0.9×10^3^ neurons/mm^3^ co-express *egr-1* and ERβ in supragranular, granular and infragranular layers of V1, respectively. These results further revealed that 29.2%±1.8 and 56.6%±3.4 of the overall neuronal population of V1 neurons expressing ERα and ERβ are engaged by visual experience. These findings suggest that although both estrogen-producing and estrogen-sensitive neurons in V1 are significantly driven by visual input, sensory experience predominantly affects the population of estrogen-producing cells.

### Estrogen-Associated Circuits in V1 Are Neurochemically Heterogeneous

**Figure 4:**
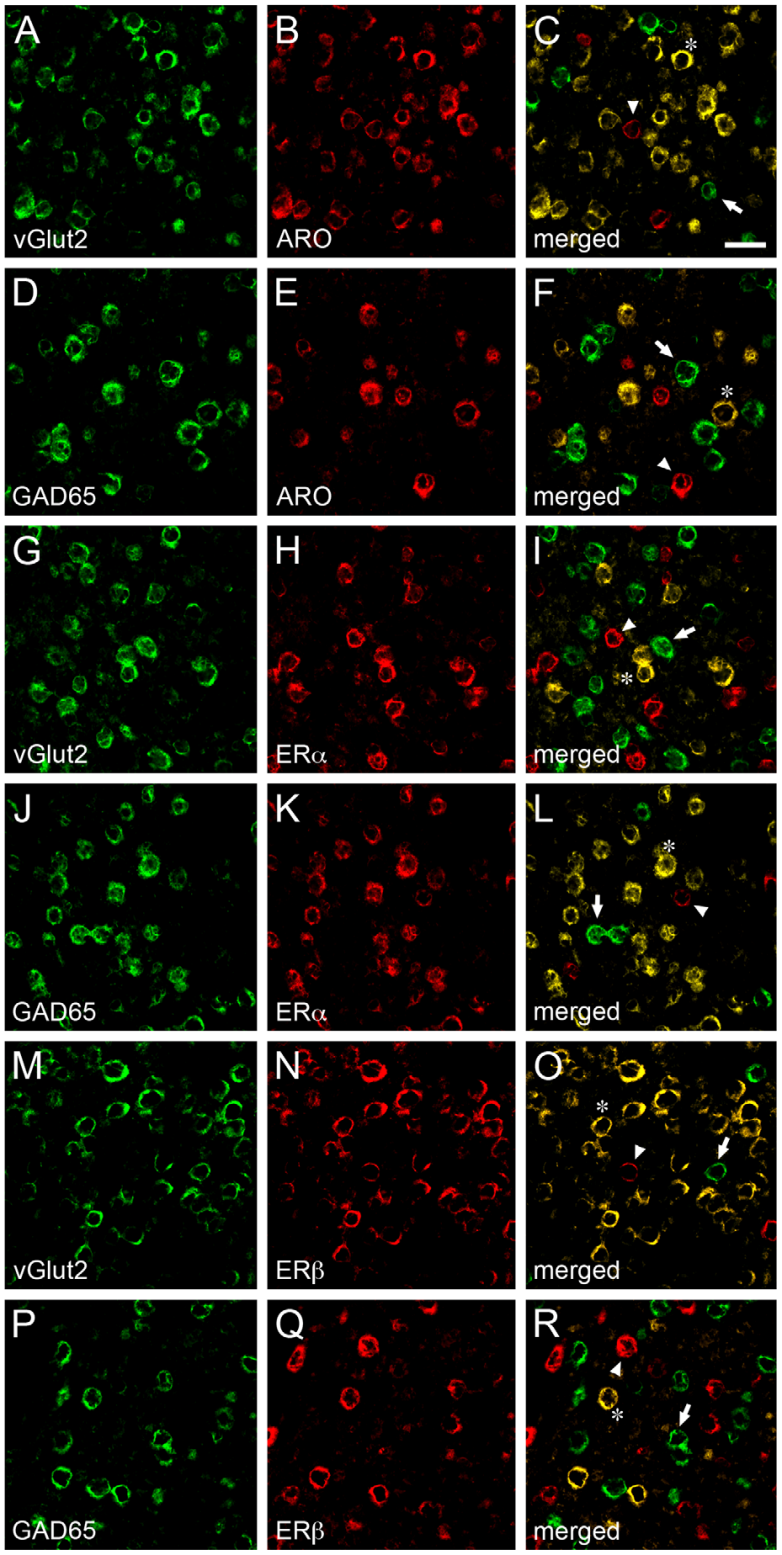
Neurochemical identity and heterogeneity of estrogen-associated circuits in V1. A–F) Images depicting representative dFISH signal in V1 for vGlut2, a marker for excitatory neurons (A) or GAD65, a marker for inhibitory neurons (D), and ARO (B, E) mRNAs. Note that ARO-positive neurons strongly co-localize with vGlut2 (C), but not GAD65 (F), indicating that estrogen-producing cells in V1 are largely excitatory neurons. G–L) Photomicrographs illustrating dFISH labeling for vGlut2 (G) or GAD65 (J), and ERα (H, K). Notably, whereas few ERα-positive neurons are excitatory (I), the vast majority of these cells co-express GAD65 (L), indicating a GABAergic phenotype. M–R) Images depicting dFISH signal for vGlut2 (M) or GAD65 (P), and ERβ (N, Q) in V1. The merged images (O, R) demonstrate that most ERβ-positive cells are excitatory, but not inhibitory, as revealed by co-localization of vGlut2 (O) and GAD65 (R), respectively. For all merged panels (right-most images in the figure plate), representative double-labeled neurons are highlighted by asterisks. Neurons that are exclusively labeled for either neurochemical cell marker, or markers for estrogen-associated circuits, are depicted by arrows and arrowheads, respectively. Scale bar = 25 µm.

We next set out to determine the neurochemical identity of estrogen-producing and estrogen-sensitive neurons in V1. To this end, we carried out double-FISH experiments combining riboprobes directed at estrogen-associated networks (ARO, ERα or ERβ) and classic markers for excitatory or inhibitory neurons – the vesicular glutamate transporter 2 (vGlut2) and the 65 kDa glutamic acid decarboxylase (GAD65), respectively (Fig. 4).

**Figure 5:**
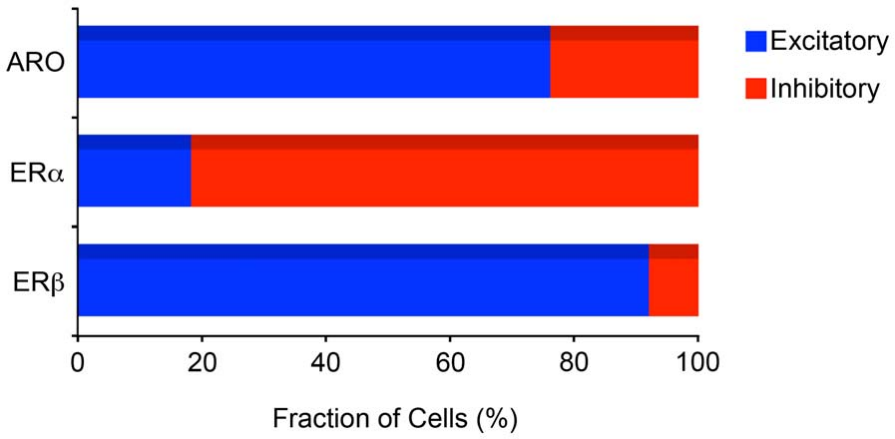
Estrogen-associated circuits in V1 are neurochemically distinct. Horizontal bar graphs expressing the percentage (± S.E.) of estrogen-producing (ARO-positive) and estrogen-sensitive (ERα and ERβ-positive) neurons that are excitatory, as revealed by co-expression of vGlut2 mRNA, or inhibitory, as revealed by co-expression of GAD65 mRNA. Note that whereas most ARO- and ERβ-positive cells are excitatory, the vast majority of neurons expressing ERα are GABAergic neurons.

We found that estrogen-producing (ARO-positive) cells are largely composed of excitatory neurons. More specifically, quantitative analysis showed that the supragranular, granular and infragranular layers of V1 displayed 18.8±0.6, 19.1±0.7 and 18.6±0.7×10^3^ neurons/mm^3^ that co-expressed ARO and vGlut2 mRNAs, respectively. In contrast, a significantly smaller fraction of ARO neurons were positive for GAD65 (6.7±0.6, 5.9±0.9 and 6.2±0.8×10^3^ neurons/mm^3^ in supragranular, granular and infragranular layers, respectively; (Fig. 4A–F). When all cortical layers were considered together, our results revealed that 76.1%±0.5 and 25.4%±1.1 of the estrogen-producing neurons in V1 (ARO-positive) were excitatory and inhibitory, respectively (Fig. 5).

Remarkably, our dFISH studies also revealed that the population of estrogen-sensitive neurons (ER-positive) is neurochemically heterogeneous. Specifically, we found that 3.3±0.7, 2.5±0.8 and 3.0±0.6×10^3^ neurons/mm^3^ co-expressed ERα and vGlut2, and 12.8±0.9, 14.1±0.8 and 13.9±0.8×10^3^ neurons/mm^3^ were double-labeled for ERα and GAD65 mRNAs in the supragranular, granular and infragranular layers of V1, respectively (Fig. 4G–L). In stark contrast, quantitative analysis revealed that whereas 19.1±0.7, 17.9±0.7 and 18.8±0.9×10^3^ neurons/mm^3^ were positive for ERβ and vGlut2 mRNAs, 2.1±0.9, 2.0±1.0 and 1.9±0.8×10^3^ neurons/mm^3^ co-expressed ERβ and GAD65 in V1's supragranular, granular and infragranular layers, respectively (Fig. 4M–R). Quantitatively, the populations of ERα- and ERβ-positive cells that were excitatory or inhibitory were significantly different from each other (p<0.001 for all layers). When considering all cortical laminae together, these findings showed that 18.2%±1.7 and 84.2%±1.8 of the ERα-expressing neurons in V1 exhibit excitatory and inhibitory phenotypes, respectively (Fig. 5). Conversely, 92.0%±2.1 and 10.1%±0.4 of the ERβ-positive neurons are composed of excitatory and inhibitory cells (Fig. 5). These findings provide direct evidence of a marked dichotomy on the neurochemical identity of ERα- versus ERβ-positive cells within V1.

### Populations of ARO- and ERβ-Positive Neurons Exhibit a Moderate Degree of Overlap

The findings above demonstrate that ERα-positive neurons are predominantly GABAergic and, therefore, represent a different neuronal population relative to ARO and ERβ-positive neurons, which are chiefly excitatory. It is unclear, however, whether or not ARO-and ERβ-positive neurons are expressed in the same cells, or different neurons. Such configurations would support models by which estrogen acts through autocrine-like and paracrine fashions, respectively. To address this issue we performed double-FISH studies for ARO and ERβ. Quantitative analysis with unbiased stereological methods revealed that 14.3±0.5, 14.8±0.8 and 16.3±0.6×10^3^ neurons/mm^3^ co-express ARO and ERβ mRNAs in the supragranular, granular and infragranular layers of V1, respectively. These observations reveal that, when considering all cortical layers together, 61.4%+5.6 of all ARO-positive neurons in V1 co-express ERβ. Overall these findings suggest that ARO and ERβ-positive neurons overlap to a moderate degree and suggest that locally-generated estrogen is positioned to affect V1 neuronal physiology through autocrine and/or paracrine fashions.

### Estrogen-Associated Networks Are Highly Stable in Response to Acute Visual Experience

To investigate if acute epochs of visual experience affect the density of estrogen-associated circuits, we subjected different groups of animals to 30 min, 1 h and 2 h of visual stimulation following overnight dark-rearing. Stereological quantification of the numerical densities of neurons positive for ARO, ERα and ERβ mRNA was carried out in brains processed for FISH.

**Figure 6:**
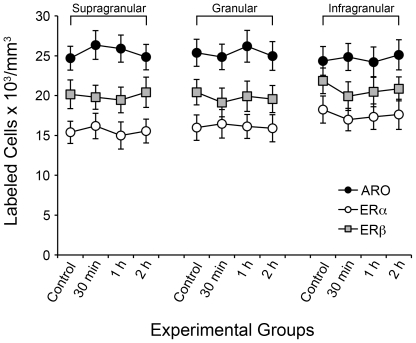
The numerical densities of ARO, ERα or ERß-positive neurons in V1 are not affected by acute visual stimulation. Line graphs depicting the mean (± S.E.) numerical densities of ARO-, ERα and ERβ-positive cells in the V1 of unstimulated (control) adult mice, as well as in animals subjected to 30 min, 1 h or 2 h of ambient light stimulation (see Methods). Data is shown for supragranular (II/III), granular (IV) and infragranular (V/VI) layers of the V1 separately. No significant differences were detected for any of the layers and experimental groups analyzed, for ARO, ERα or ERβ-positive cells.

We found that acute visual experience did not affect the population of ARO-positive cells in V1 (Fig. 6; Table 1). Likewise, acute visual stimulation did not impact the population of cells expressing either estrogen receptor across cortical layers of V1 (Fig. 6; Tables 2 and 3). Overall, the findings above indicate that acute visual experience does not affect the numerical densities of ARO- ERα- and ERβ-positive neurons and suggest that estrogen-associated circuits in V1 are highly stable to short epochs of visual stimulation.

**Table 1:** Acute sensory stimulation does not affect the numerical densities of ARO-positive cells across cortical layers of V1.

Layers/Group	Control	30 min	1 h	2 h
**Supragranular**	24.7±1.5	26.3±1.8	25.9±1.7	24.8±1.6
**Granular**	25.4±1.7	24.8±1.6	26.2±2.0	24.9±1.8
**Infragranular**	24.3±1.8	24.9±1.7	24.2±1.9	25.1±1.9

Data are expressed as mean ×10^3^ neurons/mm^3^ ± S.E. No significant differences were detected across groups for all cortical layers (all p>0.05).

**Table 2:** Acute sensory stimulation does not affect the numerical densities of ERα-positive cells across cortical layers of V1.

Layers/Group	Control	30 min	1 h	2 h
**Supragranular**	15.4±1.4	16.2±1.6	14.9±1.7	15.5±1.5
**Granular**	15.9±1.6	16.4±1.8	16.1±1.5	15.9±1.7
**Infragranular**	18.2±1.7	17.0±1.4	17.3±1.6	17.6±1.9

Data are expressed as mean ×10^3^ neurons/mm^3^ ± S.E. No significant differences were detected across groups for all cortical layers (all p>0.05).

**Table 3:** Acute sensory stimulation does not affect the numerical densities of ERβ-positive cells across cortical layers of V1.

Layers/Group	Control	30 min	1 h	2 h
**Supragranular**	20.1±1.8	19.8±1.5	19.4±1.6	20.4±1.9
**Granular**	20.4±1.6	19.1±1.8	19.9±1.9	19.6±1.7
**Infragranular**	21.8±1.6	19.9±1.8	20.4±1.9	20.8±1.5

Data are expressed as mean ×10^3^ neurons/mm^3^ ± S.E. No significant differences were detected across groups for all cortical layers (all p>0.05).

### Estrogen-Associated Circuits Are Not Affected by Chronic Visual Deprivation

Although acute visual stimulation does not influence the density of estrogen-producing and estrogen-responsive neurons, it is possible that chronic (long-term) changes in visual experience affect the constitution of estrogen-associated circuits. To directly investigate this question, we raised a group of mice from birth to adulthood in complete darkness. We then quantitatively compared the distribution of ARO, ERα and ERβ-positive cells in the V1 of these chronically light-deprived mice against that of age-matched, normally-raised controls.

**Figure 7:**
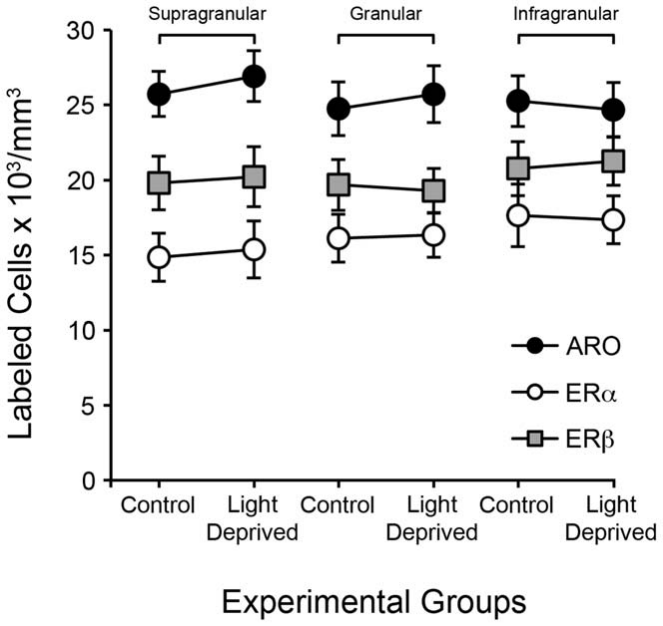
Chronic visual deprivation, from birth to adulthood, does not affect the density of estrogen-associated circuits in V1. Shown are the mean numerical densities (± S.E.) of cells that are positive for ARO-, ERα- and ERβ-positive in the V1 of control (normally-raised) and light-deprived adult mice. Data is shown for supragranular, granular and infragranular layers separately. Light-deprived animals were born and kept in complete darkness until adulthood (see Methods). No differences were detected for ARO-, ERα- or ERβ, for any cortical layers, when comparing control and chronically light-deprived animals.

Surprisingly, our results showed that chronic visual deprivation does not affect the density of ARO-positive neurons in V1 (Fig. 7; Table 4). Likewise, animals that were dark-reared from birth exhibited numerical densities of ERα and ERβ-positive cells across cortical layers of V1 that were not significantly different from those observed in normally-reared controls (Fig. 7; Tables 5 and 6, respectively).

**Table 4:** Chronic visual deprivation does not affect the numerical densities of ARO-positive cells across cortical layers of V1.

Layers/Group	Dark-Reared	Normally-Reared
**Supragranular**	26.9±1.7	25.7±1.5
**Granular**	25.7±1.9	24.8±1.8
**Infragranular**	24.7±1.8	25.3±1.7

Data are expressed as mean ×10^3^ neurons/mm^3^ ± S.E. No significant differences were detected across groups for all cortical layers (all p>0.05).

**Table 5:** Chronic visual deprivation does not affect the numerical densities of ERα-positive cells across cortical layers of V1.

Layers/Group	Dark-Reared	Normally-Reared
**Supragranular**	15.3±1.9	14.8±1.6
**Granular**	16.3±1.5	16.1±1.6
**Infragranular**	17.3±1.6	17.5±2.1

Data are expressed as mean ×10^3^ neurons/mm^3^ ± S.E. No significant differences were detected across groups for all cortical layers (all p>0.05).

**Table 6:** Chronic visual deprivation does not affect the numerical densities of ERβ-positive cells across cortical layers of V1.

Layers/Group	Dark-Reared	Normally-Reared
**Supragranular**	20.2±2.0	19.7±1.8
**Granular**	19.3±1.5	19.6±1.7
**Infragranular**	21.2±1.6	20.7±1.8

Data are expressed as mean ×10^3^ neurons/mm^3^ ± S.E. No significant differences were detected across groups for all cortical layers (all p>0.05).

Our results show that chronic visual deprivation throughout post-natal development does not affect the numerical densities of estrogen-associated circuits. These findings suggest that visual experience is not required for the adequate implementation and maintenance of estrogen-sensitive or estrogen-responsive circuits in V1.

## Discussion

Over the past decade accumulating evidence has pointed towards a strong relationship between changes in the circulating levels of the classic steroid hormone E2 and the function of sensory systems, including the visual system [4], [9], [10], [11], [12], [13], [14], [15], [27], [28]. Recently, our group provided the first direct evidence that brain-derived E2, more specifically E2 produced by central auditory neurons, directly regulates hearing-driven auditory responses in the awake brain, in real-time, by regulating the strength of local inhibitory transmission [17]. We further showed that this brain-generated E2 acts to enhance the coding efficiency of auditory neurons to optimize the neural and behavioral discrimination of acoustic cues [29]. These recent findings raised the possibility that this novel sensory-neuroendocrine interaction may be a general property of sensory systems and, consequently, extend beyond the auditory system.

Here we investigated whether the previously shown relationship between E2 levels and visual function may have an underlying neural basis that is analogous to our earlier findings in the auditory forebrain – namely, the presence of robust estrogen-associated networks embedded within V1 circuitry. Remarkably, our results directly demonstrate that the mouse V1 is a site of putative production and sensitivity to estrogens. The majority of V1 neurons express ARO (estrogen-synthase), a marker of estrogen-producing neurons. Additionally, a significant neuronal population also expresses the classic estrogen receptors ERα and ERβ. We further uncovered the neurochemical identity and a heterogeneity in estrogen-associated networks in V1; whereas ARO- and ERβ-positive cells are primarily excitatory, most ERα-positive cells are GABAergic neurons. Furthermore, we discovered that most (but not all) neurons in V1 co-express ARO- and ERβ, suggesting that locally-generated estrogen may affect visual cortical processing through autocrine and/or paracrine mechanisms. Our results also show that estrogen-producing, and to a lesser extent estrogen-sensitive neurons, are activated by visual experience in freely-behaving animals and that acute or chronic changes in visual experience do not affect the distribution of the components of estrogen-associated circuits in V1. These results indicate that estrogen circuits in V1 are implemented and maintained in an experience-independent fashion, but are directly engaged by sensory input in behaving animals.

Together, these observations raise the possibility that the cortical processing of visual stimuli is likely to be subject to modulation by brain-derived E2, as has been described in the auditory system [4], [16], [17], [29]. To our knowledge, our findings provide the first quantitative demonstration of robust estrogen-associated circuits in the adult mammalian V1 and indicate that brain-derived estrogenic modulation of sensory processing may not be constrained to central auditory circuits, but rather may generalize to other sensory modalities, including vision. As such, brain-generated E2 may constitute a key neuromodulatory component influencing the operational framework of sensory systems in the adult vertebrate brain.

### Influences of Estrogen on Visual Cortical Processing

The presence of estrogen-producing and -sensitive neurons in V1 is congruent with recent findings obtained in Esr2 (ERβ) bacterial artificial chromosome transgenic mice [30], a model that overcomes known technical limitations associated with antibodies directed against estrogen receptors [24], [30]. Our findings suggest roles for local estrogen signaling in shaping cortical visual responses. While these effects could involve the classical genomic pathway through ER signaling-induced transcriptional changes, fast non-genomic signaling has been described to play important roles in the central nervous system. For instance, in the auditory system, E2 levels are markedly and rapidly regulated by sensory experience in freely-behaving animals [16]. The fact that visual stimulation engages primarily estrogen-producing neurons suggests that a similar mechanism may be implemented within the visual cortex and raise the intriguing possibility that E2 levels may rapidly oscillate in V1 as a function of visual experience.

Our findings also uncovered the neurochemical identity of estrogen-producing and estrogen-sensitive cells in V1, and showed that these populations are phenotypically distinct. Whereas ARO- and ERβ-positive cells are primarily excitatory, ERα-positive neurons are largely inhibitory. These findings have significant implications for the potential roles of E2 in shaping receptive field properties of V1 neurons. For example, local E2-produced by ARO-positive cells may directly modulate excitatory spatio-temporal processing and intracortical computations in V1 via ERβ receptors. In contrast, E2 is well positioned to selectively modulate local inhibition through activation of ERα receptors, and consequently influence cell thresholds, lateral inhibition, the sharpness of orientation tuning and direction selectivity, all of which are functional properties previously shown to be directly regulated by GABAergic transmission in V1 [31], [32], [33], [34]. Future studies coupling neurophysiological recordings and local intracerebral pharmacological manipulations in the awake brain should provide direct tests for these possibilities.

### Potential Mechanisms Underlying Estrogenic Influences in V1's Neuronal Physiology

In the context of real-time sensory processing, significant interest has been centered on uncovering the mechanistic bases of estrogen's rapid (non-genomic) effects in neuronal physiology. Most of our current understanding about how E2 rapidly shapes neuronal responses has derived from findings obtained in the hippocampus. For example, the magnitude of AMPA, kainate and NMDA receptor EPSCs are markedly enhanced by E2 [1], [35], [36], [37], [38], [39]. Recent findings also show that E2 regulates pre-synaptic glutamate release via an ERβ-dependent mechanism [40]. In central auditory neurons, our group showed that E2 selectively suppresses GABAergic transmission through a pre-synaptic mechanism [17]. Although a systematic evaluation will be required to determine the precise mechanisms through which E2 may rapidly influence the physiology of visual cortical neurons, it is plausible, and perhaps highly likely, that E2 may affect fast neurotransmission in V1, based on these earlier findings. Slower effects of E2 on neurotransmitter systems have also been previously reported for an array of brain areas, and could also occur in V1. For instance, the expression of GABA receptors has been shown to be regulated by E2, suggesting that this hormone may also exert slower effects on receptor composition and/or distribution, and consequently influence post-synaptic responses in V1 [41], [42], [43], [44].

### Estrogenic Influences on Visual Cortical Plasticity

Estrogenic signaling has also been tied to synaptic plasticity. Fluctuations in E2 levels that occur during different phases of the estrous cycle correlate with oscillations in dendritic spine density in the rodent hippocampus and sensorimotor cortex, and alters spatial learning and memory in both animals and humans [45], [46], [47], [48], [49]. Moreover, E2 infusion systemically or into the hippocampus enhances hippocampal-dependent memory [50], [51]. Consistent with this view, E2 rapidly enhances synaptic plasticity in the hippocampus, more specifically long-term potentiation of post-synaptic responses [52], [53], and modulates dendritic spine morphogenesis [54], [55], [56], [57]. It is interesting to note that E2's effects on hippocampal synaptic plasticity appear to be mediated by ERβ receptors, which activate the extracellular signal-related kinase/mitogen-activated protein kinase (MAPK) pathway, a biochemical cascade repeatedly implicated in synaptic plasticity, learning and memory formation [17], [19], [58], [59]. Our group has recently shown that E2 produced by central auditory neurons is both necessary and sufficient to drive multiple MAPK-dependent plasticity-associated genes [17], all of which are robustly expressed in V1 as a result of visual experience [19], [20], [21], [22], [23], [60]. The prominent presence of ARO and ERs in visual cortical neurons makes it highly likely that estrogenic signaling in the adult visual cortex may also contribute to plastic processes elicited by vision.

### Activity-Dependent Estrogen Signaling in V1

Our discoveries that the majority of neurons that expressed ARO, as well as neurons that expressed ERs, were sensitive to visual stimulation suggest that local E2 production, and to a lesser extent E2 sensitivity, may be altered in an activity-dependent manner by sensory stimuli. However, our experiments did not detect changes in the distribution of ARO- or ER-positive cells following acute light exposure in dark-adapted animals, or as a result of chronic sensory deprivation in the form of dark rearing from birth to adulthood. These findings suggest that the development and implementation of estrogen-associated networks is independent of sensory experience, and consequently, is likely to be activity-independent or to exclusively rely on spontaneous thalamo-cortical activity [61], [62], [63]. Since dark rearing has profound effects on the development of both excitatory and inhibitory circuits in the visual cortex [64], [65], our data also indicate that the development and maintenance of estrogenic signaling in V1 is robust in the presence of altered visual activity and, consequently, such circuits are highly stable. These experiments do not rule out the possibility, however, that acute or chronic changes in visual experience may affects the transcriptional activity of the genes encoding ARO and/or ERs. Our results also do not speak to changes in ARO or ERs that may occur on a translational or post-translational level. In fact, fast post-translational modulation of ARO activity has been described in the hypothalamus and is elicited by activity and calcium-dependent phosphorylation of this enzyme [66], [67], [68], [69]. Whether such mechanisms also operate in sensory cortical areas is unknown. In the future it will be important to develop tools that will allow quantification of potential rapid post-translational modifications of ARO and ERs, as well as methods of measuring local E2 concentrations in the visual cortex of freely-behaving animals, as has been done for other species [16]. These mechanisms could provide fast regulation of E2 levels, in an activity-dependent manner, and consequently position E2 as a powerful neuromodulator of visual function.

### Summary, Limitations and Future Studies

We demonstrate that the mouse visual cortex has all the necessary elements to implement local estrogenic signaling during visual cortical processing. Congruent with this view, we also showed that visual experience engages estrogen-associated circuits in V1 and, consequently, may impact local E2 production and sensitivity. Our data demonstrates that estrogen-associated circuits in V1 are neurochemically distinct and, therefore, are well positioned to influence receptive field tuning properties, and cortical processing, in multiple ways. Finally, we show that acute or chronic changes in visual experience do not appear to affect the organization of estrogen-associated circuits in V1, suggesting that stability of this network may be tightly regulated to consistently influence visual processing.

Although our findings provide direct evidence for the robust presence of estrogen-associated circuits in the adult V1, a sensory area that has been largely ignored in relation to neurohormonal action, future studies will be required to determine additional anatomical and functional features of this sensory-neuroendocrine interaction. For instance, it will be important to determine whether or not the protein products of ARO and ERs are expressed at the light and electron microscopy level. It will also be important to establish in future studies if, and to what extent, sensory input modulates ARO expression and/or activity. This possibility would be congruent with earlier findings suggesting that excitatory neurotransmitters directly modulate ARO activity and that excitotoxic activity that results from stroke regulates estrogen-mediated neuroprotection [66], [67], [68], [69], [70], [71]. Finally, considering the overlap between ARO- and ER-positive neurons in V1, future efforts should be aimed at determining if activation of ERs modulates ARO activity, suggestive of an autocrine-like fashion.

Given recent studies showing that fast, non-genomic, local estrogenic signaling modulates critical aspects of central auditory processing in the awake vertebrate brain, our data suggest that similar mechanisms are likely to play profound roles in visual cortical function. Thus, our findings are highly suggestive that brain-generated estrogenic modulation of sensory processing may not be an exclusive feature of auditory circuits, but rather may generalize to other sensory modalities, including vision. Consequently, our findings also suggest that brain-generated E2 may constitute a fundamental component of the operational framework of sensory circuits in the vertebrate brain.
